# Novel alkaloids from the fire ant, *Solenopsis geminata*

**DOI:** 10.1007/s00114-022-01786-w

**Published:** 2022-01-27

**Authors:** Robert K. Vander Meer, Satya P. Chinta, Tappey H. Jones

**Affiliations:** 1 USDA/ARS, CMAVE, 1600 SW 23rd Drive, Gainesville, FL 32608 USA; 2 Foresight Science and Technology, Hopkinton, MA 01748 USA; 3 Department of Chemistry, Virginia Military Institute, Lexington, VA 24450 USA

**Keywords:** *Solenopsis geminata*, Alkaloids, Fire ant, Venom, Piperidine, Pyridine

## Abstract

South American fire ants, *Solenopsis richteri* and *Solenopsis invicta*, were accidently introduced into the southern USA in the 1900s and 1930s, respectively. The rapid spread and high population densities of *S. invicta*, and its potent sting, resulted in broad economic impacts and a variety of research efforts. In the 1970s, their venom alkaloids were identified as a complex blend of *trans*-2-methyl-6-alkyl- and alkenyl-piperidines. *Solenopsis geminata* is a worldwide tramp species but a native of the southern coastal regions of the USA. It was found to only produce *cis*- and *trans*-2-methyl-6-undecyl-piperidines. These alkaloids were considered the *Solenopsis* ancestral alkaloid profile since they were identified from female sexuals (potential queens) of all *Solenopsis* species in South and North America. The dramatic modification of alkaloids in *Solenopsis invicta* was attributed to their response to evolutionary pressure and the lack of change in *S. geminata* alkaloids due to no response to evolutionary pressure. Here we report the unexpected discovery of 6-undecyl-pyridine, 2-methyl-6-undecyl-pyridine and 2-methyl-6-(1)-undecenyl-pyridine as components of *S. geminata* worker venom, suggesting that *S. geminata* like its South American relatives have responded to evolutionary pressures. Our results will stimulate future research on *S. geminata* populations throughout the tropical/subtropical world.

## Introduction

The fire ants, *Solenopsis geminata* and *Solenopsis xyloni* (members of the *S. geminata* species complex, Pitts et al. 2018), are native to the southern USA and have a long history of human interactions. While they have a significant sting, it was the accidental introduction of two South American fire ants into the southern USA, *Solenopsis richteri* (1920s) and especially *Solenopsis invicta* (1930s) (*Solenopsis saevissima* species complex, Pitts et al. 2018), which generated widespread attention. High population densities of *Solenopsis invicta* and a propensity for disturbed habitats led to frequent human interactions and broad economic impact. Five *trans*-2-methyl-6-alkyl-(C_11:0_, C_13:0_, C_15:0_) and alkenyl-(C_13:1,_ C_15:1_)-piperidines were identified from *S. invicta* worker venom (MacConnell et al. 1971). Venom alkaloid research was extended to workers of the native fire ants, *S. geminata* and *S. xyloni*. They produce a mixture of *cis*- and *trans*-2-methyl-6-undecyl-piperidines (Brand et al. 1972, Fig. 1, compounds 2 and 4), in contrast to the variety of piperidine alkaloids that dominate the two invasive species. Since all female sexual forms (future queens) associated with the *S. geminata* and *S. saevissima* species complexes produce *cis*- and *trans*-2-methyl-6-undecyl-piperidines, these two alkaloids were considered the ancestral type. It was thought *S. invicta* and *S. richteri* workers evolved beyond the undecyl-piperidines, while *S. geminata* species complex workers maintained the ancestral alkaloid blend (Brand, 1978).

**Fig. 1:**
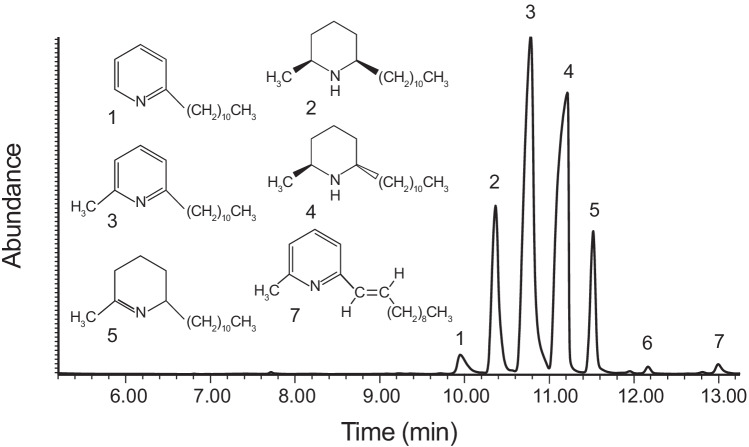
The total ion chromatogram is shown for an extract of *S. geminata* workers (Gainesville, FL) immersed in hexane for 24 h. The seven peaks were demonstrated to be composed of single components rather than mixtures of co-eluting compounds (MassWorks, Cerno Bioscience, https://cernobioscience.com). The structures for six of the seven alkaloids were determined by mass spectral fragmentation patterns, synthesis, and co-injection and are inserted into the chromatogram

Alkaloid use in chemotaxonomy was illustrated by Brand et al. (1973), in that *S. xyloni* and *S. geminata* workers could be identified by their *cis/trans* 2-methyl-6-undecyl-piperidine ratio. A broad survey of *Solenopsis* venom alkaloids in North and South America also supported alkaloid use in chemotaxonomy (MacConnell et al. 1976). Also, Brand et al. (1972) found detectable amounts of 2-methyl-6-undecyl-Δ^1,2^-piperideine (see Fig. 1, compound (5), in *S. xyloni* venom) but suggested it was a precursor to the major piperidine venom components, as later verified by Leclercq et al. (1996). A reproductively viable and morphologically cryptic hybrid between *S. invicta* and *S. richteri* was initially detected based on their venom alkaloid profiles (Vander Meer and Lofgren, 1985).

Piperideine alkaloids from *S. invicta* were reported as minor components by two research groups (Chen et al. 2009; Chen and Fadamiro, 2009). Both groups suggested that the piperideines likely functioned as piperidine precursors (Leclercq et al. 1996). A focused study of *S. geminata* venom alkaloids (Shi et al. 2015) isolated the expected *cis*- and *trans*-2-methyl-6-undecyl-piperidines as the major components. Pyridine alkaloids were not reported from *Solenopsis* venom until Chen et al. (2019) described trace amounts of ten pyridines from workers of *S. invicta*, *S. richteri*, and their hybrid. The identified pyridines were each < 1 ng/ant in both species and their hybrid. For perspective, each *S. invicta* worker produces about 18 μg of venom alkaloids (Haight and Tschinkel 2003).

*Solenopsis geminata* alkaloids were well established as *cis*- and *trans*-2-methyl-6-undecyl-piperidines when in 2019 three worker ants intercepted at a port in Hawaii, identified as *Solenopsis*, were sent to our facility to determine if they were *S. invicta*. Molecular analyses (mitochondrial *COX1* gene) classified the ants as *S. geminata*. Chemical analysis unambiguously identified two major components as 2-methyl-6-undecyl-pyridine and *trans*-2-methyl-6-undecyl-piperidine. The normally dominant cis-2-methyl-6-undecyl-piperidine was a minor component (Ascunce et. al. 2021). This surprising result was the driving force for reinvestigation of *S. geminata* venom alkaloids.

Here we report the unexpected discovery of 6-undecyl-pyridine, 2-methyl-6-undecyl-pyridine, and 2-methyl-6-(1)-undecenyl-pyridine (compounds 1, 3, and 7 in Fig. 1) as new alkaloid components of *S. geminata* venom from Gainesville, FL, USA.

## Methods

### Sampling

**Fig. 2:**
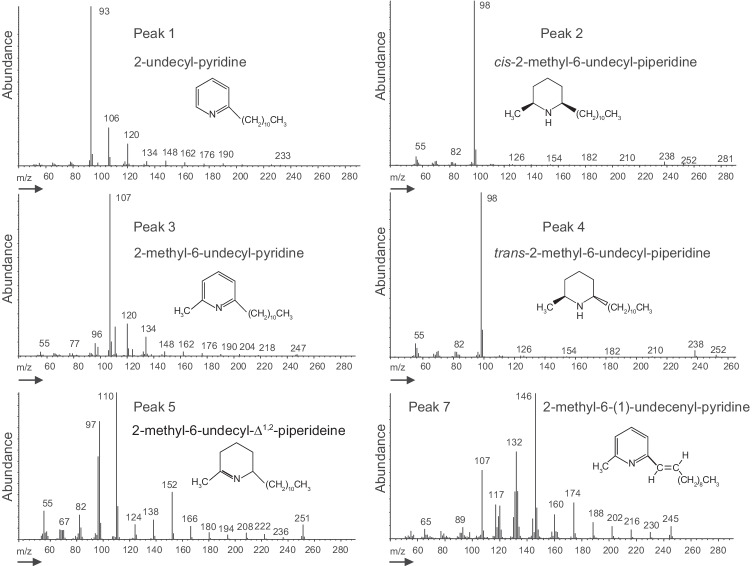
The mass spectral fragmentation patterns and associated structures are shown for each of the 6 identified *S. geminata* alkaloid components from an extract of *S. geminata* workers (Gainesville, FL) immersed in hexane for 24 h. The structures for six of the seven alkaloids were determined by mass spectral fragmentation patterns, synthesis, and co-injection

**Fig. 3:**
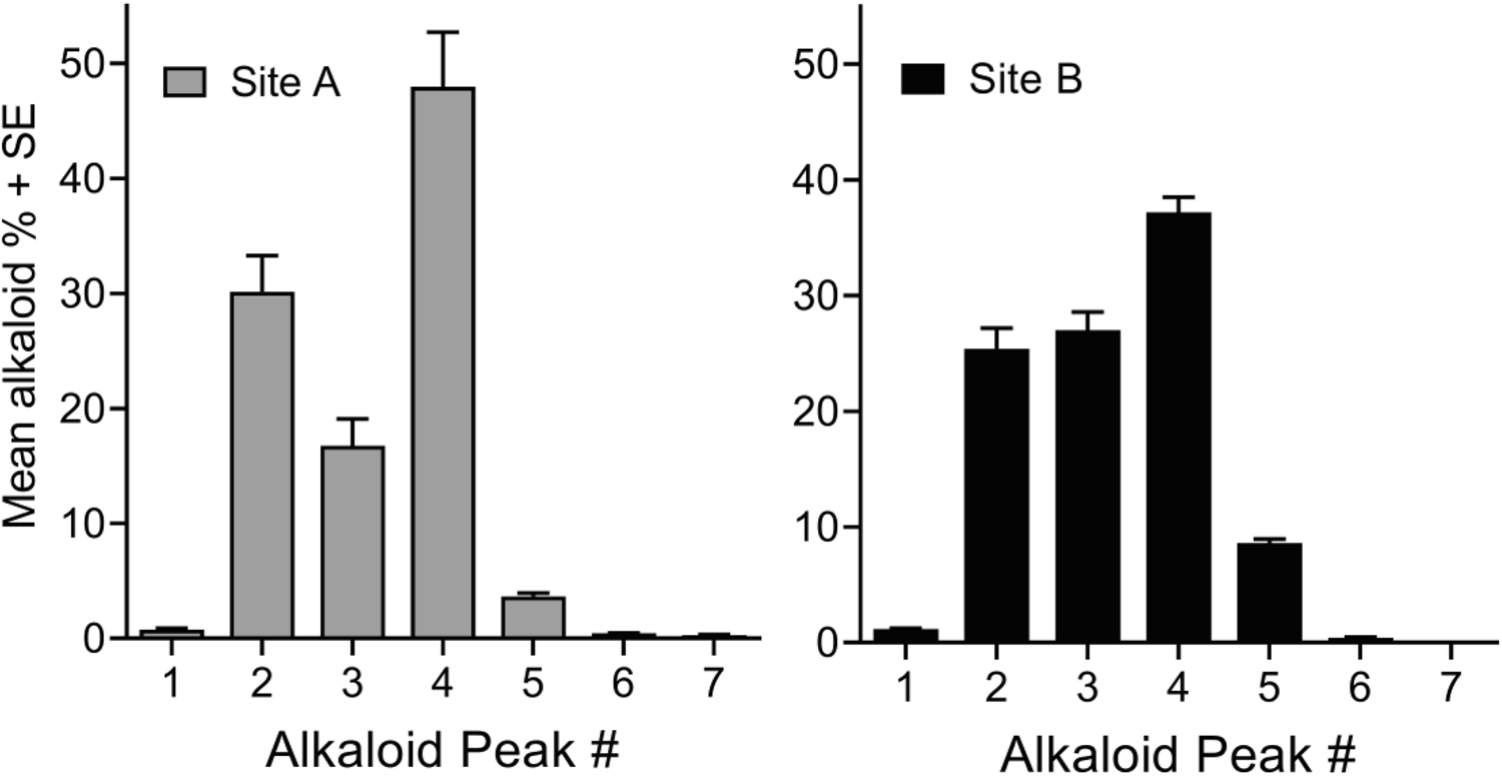
The mean + SE of the percent composition for the 7 alkaloid components based on the integration values for each sample (total ion chromatogram, TIC: (1) collection site A, 2015, *n* = 9, and (2) collection site B, 2019. Locations A and B are approximately 25 km apart. Each replicate represents a unique *S. geminata* colony

The tropical fire ant, *S. geminata*, inhabits the southern tier of the USA and can be found in the Gainesville, FL, area, normally in dry, well-drained areas that are less likely to be occupied by the invasive fire ant, *S. invicta*. *Solenopsis geminata* is unique in that it can be readily differentiated from other *Solenopsis* species by the presence of a worker caste that has a disproportionately large head. All samples of *S. geminata* workers (20–50) were collected from individual colonies (replicates) and placed in a vial containing enough hexane (Fisher Optiva, Cole Palmer, Vernon Hills, IL) to cover the ants. After 24 h, the hexane was pipetted into a clean vial in preparation for GC–MS analysis to determine tentative component identification and relative component percentages. Collection site A was sampled in 2015. At that time, cuticular hydrocarbon patterns were of prime interest since the alkaloids were assumed to be *cis*- and *trans*-2-methyl-6-undecylpiperidines. The GC–MS temperature program targeted separation of cuticular hydrocarbons. The two major alkaloid peaks (2) and (4), Fig. 1, matched the expected cis- and trans-piperidine fragmentation pattern (base fragment at *m/e* = 98, see Fig. 2). The alkaloids are antibiotic and very stable and have been recovered after decades of room temperature storage (see method for synthetic component 3). In view of the Hawaii results (Ascunce et al., 2021), the 2015 samples (site A, *n* = 9) were reconstituted in hexane, vortexed, and analyzed using GC–MS program b. Fragmentation patterns (Fig. 2) were compared to synthetic standards. Components 1 to 5 and 7 (Fig. 1) were identified. The TIC (total ion chromatogram) was used to integrate the areas under each peak, and the peak percent composition was calculated. Twelve additional samples were collected and analyzed in 2019 from Gainesville, FL, site B, which was approximately 25 km from site A. Graphical representations of alkaloid peak proportions were prepared (Fig. 3, site A and site B) using GraphPad Prism, version 9.01 (GraphPad Software Inc., San Diego, CA).

### Gas chromatograph-mass spectroscopy (GC–MS) analysis

Mass spectral data were obtained from (a) Shimadzu QP-2020 GC–MS (Palo Alto, CA) equipped with an RTX-5 ms, 30 m × 0.25 mm i.d. column. The instrument was temperature programmed from 60 to 250 °C at 10°/min and held there for 20 min (Virginia Military Institute, all synthetic samples) or (b) Agilent Intuvo 9000 GC system (Santa Clara, CA) equipped with an HP-5 MS ultra-inert nonpolar column, 30 m × 0.25 m i.d. column, coupled to a 5977 B mass spectral detector and a MassHunter Data Acquisition Workstation version 10.0.368 (Santa Clara, CA). Injector temperature was set at 250 °C. The oven temperature was programmed at 40 °C for 2 min and then to 285 °C at 5 °C/min, followed by a 10 min hold at 285 °C (USDA, Gainesville, Florida). Agilent’s MassHunter and NIST MS Library Database (2020) was used to compare venom component mass spectral fragmentation signatures (see Fig. 2). MassWorks software (Cerno Bioscience, https://cernobioscience.com/) determined the purity of each peak and their retention index (RI) relative to a series of normal hydrocarbons.

## Results

### Identification of compounds 1–5 and 7

See Fig. 2 for the mass spectral fragmentation pattern of each compound.

### 2-undecyl-pyridine (1)

Venom component 1 (Fig. 1) was tentatively identified as 2-undecyl-pyridine through a search and match of the NIST (2020) mass spectrum library. To verify the structure assignment, the known compound was synthesized by alkylation of 2-picolyl magnesium bromide with 1-bromodecane (Proffe and Linke, 1960). The synthetic product was distilled by Kugelrohr distillation and had a mass spectral fragmentation pattern that matched the reported fragmentation pattern (Heller et al. 2002) for 2**-**undecyl-pyridine (1). This pattern was identical to that of natural *S. geminata* component (1). In addition, synthetic 2-undecyl-pyridine co-eluted with natural component (1) when co-injected on the GC–MS, verifying the structural assignment.

### cis-2-methyl-6-undecyl-piperidine (2), 2-methyl-6-undecyl-pyridine (3), and trans-2-methyl-6-undecyl-piperidine (4)

These venom components are known compounds and were synthesized by methods described in MacConnell et al. (1971). Component (3) was distilled from an original 50-year-old sample synthesized by MacConnell et al. (1971), a testament to the stability of the pyridines. GC–MS retention times, co-injections, and matched fragmentation patterns confirmed the assigned structures of venom components, (2), (3), and (4).

### 2-methyl-6-undecyl-Δ^1,2^-piperideine (5)

Component (5) was synthesized by the method described by Brand et al. (1972). The known fragmentation pattern for compound (5) and the other piperideine isomer, 2-methyl-6-undecyl-Δ^1,6^-piperideine, are distinctive (Brand et al. 1972). The fragmentation pattern of *S. geminata* component (5), Fig. 2, matched the fragmentation pattern of the 2-methyl-6-undecyl-Δ^1,2^-piperideine (5). Synthetic component (5) co-injected with the natural extract, co-eluted with tentatively identified component 5, confirming its structure.

### 2-methyl-6-(1)-undecenyl-pyridine (7)

Proposed component (7) (see Fig. 1) is a known compound and was prepared as described by Okuma et al. (2003) via a Wittig reaction of decyltriphenylphosphonium bromide with 6-methyl-2-pyridine-carboxyaldehyde, using DBU (1,8-diazabicyclo-[5.4.0]-undec-7-ene) as a base. The products of this reaction were a 1:1 mixture of *Z*- and *E*-2-methyl-6-(1)-undecenyl-pyridine. The mass spectrum (see Fig. 2) and retention time of the trans-(*E*) isomer matched those of component (7) from *S. geminata*. Co-injected *E*-2-methyl-6-(1)-undecenyl-pyridine co-eluted with component (7) in a natural *S. geminata* sample, confirming the structural assignment.

The retention index (RI) was calculated for the seven alkaloid components (Fig. 1) via MassWorks (Cerno Bioscience, https://cernobioscience.com): (1) = 1815, (2) = 1835, (3) = 1854, (4) = 1874, (5) = 1891, (6) = 1928, and (7) = 1959. The first five alkaloids elute in between C_18_ and C_19_ normal hydrocarbons.

The GC–MS data provided tentative identification of Fig. 1 peaks: (1) 2-undecyl-pyridine, (2) *cis*-2-methyl-6-undecyl-piperidene, (3) 2-methyl-6-undecyl-pyridine, (4) *trans*-2-methyl-6-undecyl-piperidene, (5) 2-methyl-6-undecyl-Δ^1,2^-piperideine, and (7) 2-methyl-6-(1)-undecenyl-pyridine. Peaks (2) and (4) are the cis- and trans-disubstituted major components reported early on for *S. geminata* (see “Introduction”). Component 5 was previously reported as minor components from *S. geminata*. Alkaloid components (1), (3), and (7) are known compounds but are reported here for the first time from *Solenopsis geminata*. Components (1) and (7) are reported for the first time from *Solenopsis* ants. Component 6 had a molecular ion at *M* +  = 263, and its mass spectrum suggested an N, 2-dimethyl-6-undecyl-dihydropyridine. The small amount present relative to the other alkaloids prevented further characterization. The relative abundance (mean + SE) of the 7 alkaloid components (Fig. 1) from collection site A, *n* = 9, and site B, *n* = 12, is shown in Fig. 3. Replicates represent *S. geminata* worker samples collected from different colonies located at the collection sites.

## Discussion

The driving force for this research was the unexpected identification of 2-methyl-6-undecyl-pyridine, Fig. 1, peak (3) as the major component from three *S. geminata* ants intercepted by the State of Hawaii quarantine officials (Ascunce et al., 2021). This discovery was contrary to the literature that over four decades reinforced *cis*- and *trans*-2-methyl-6-undecyl-piperidines as the major *S. geminata* venom alkaloids (Brand et al. 1972 to Shi et al. 2015). Prior to the 1980s, packed columns were not capable of separating the pyridines and piperidines reported here, e.g., MacConnell et al. (1976). Lai et al. (2009) investigated seasonal and caste changes in *S. geminata* worker venom from hexane extracts analyzed via GC–MS with a capillary column. They used a very short temperature program (8 min) but used single ion monitoring (SIM), m/z 98, to generate data specific to the piperidines (see Fig. 2). The pyridines and piperideines do not have an m/z 98 fragment (Fig. 2) and would be excluded. Shi et al. (2015) applied a special silica gel gravity column separation method to isolate piperidines from a total *S. geminata* extract (from Gainesville, FL) prior to GC–MS analysis. They did not identify any pyridines. Our slow GC temperature ramp led to baseline separation and identification of the six alkaloids presented here.

Our results increase the complexity of *S. geminata* alkaloids, suggesting that *S. geminata* workers have indeed responded to evolutionary pressure with alkaloid diversity more complicated than the ancestral cis/trans 2-methyl-6-undecyl-piperidines found in *Solenopsis* female sexuals. What makes our results especially intriguing is that *S. geminata* occupied coastal areas of the southern USA, and with the advent of worldwide trade routes in the mid-sixteenth century, *S. geminata* accidentally hitchhiked to many tropical/subtropical areas of the world (Wetterer 2011; Gotzek et al. 2015). The ecological habitats that *S. geminata* found itself in and survived in are extraordinarily diverse, e.g., Senegal, Canary Islands, Madagascar, Australia, Fiji, Gilbert Islands, India, Laos, and Italy (Wetterer 2011). The specific focus for this paper is on *S. geminata* in the USA and specifically Florida, but the results will be of interest to researchers throughout the tropical/subtropical areas of the world where *S. geminata* is found.

## Data Availability

The data sets generated during and/or analyzed during the current study are available from the corresponding author on reasonable request.

## Code availability

NA.
